# Active Surveillance of Low-Risk Papillary Microcarcinoma of the Thyroid: Kuma Hospital Protocol and Its Outcomes

**DOI:** 10.1089/ve.2016.0073

**Published:** 2016-09-14

**Authors:** Akira Miyauchi

**Affiliations:** Center for Excellence in Thyroid Care, Kuma Hospital, Kobe, Japan.

**Keywords:** thyroid cancer, papillary microcarcinoma, active surveillance, immediate surgery, unfavorable event

## Abstract

Many researchers have reported rapid increase in the incidence of thyroid cancer in many countries including the United States and Korea. The increase was mostly because of the increase in detection of small papillary carcinoma, and mortality rates from thyroid cancer remained stable. They suggested overdiagnosis and overtreatment of small papillary carcinoma. In Kuma Hospital, we have been conducting active surveillance of low-risk papillary carcinoma according to Miyauchi's proposal in 1993. This video reports the outcomes of our clinical trial according to the Kuma Hospital Protocol, showing that active surveillance is much better than immediate surgery for the patients with this indolent cancer.

No competing financial interests exist.

Runtime of video: 12 mins

## Graphical abstract

**Figure:**
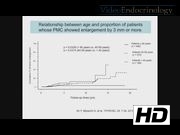

**Figure:**
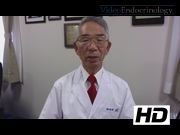

**Figure:**
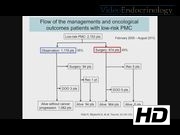

## Video

## Files

**PDF:** 

**QT:** 

**HD:** 

